# On the Consequences of Purging and Linkage on Fitness and Genetic Diversity

**DOI:** 10.1534/g3.115.023184

**Published:** 2015-11-09

**Authors:** Diego Bersabé, Armando Caballero, Andrés Pérez-Figueroa, Aurora García-Dorado

**Affiliations:** * Departamento de Genética, Facultad de Biología, Universidad Complutense, 28040 Madrid, Spain; † Departamento de Bioquímica, Genética e Inmunología, Facultad de Biología, Universidad de Vigo, 36310 Vigo (Pontevedra), Spain

**Keywords:** inbreeding load, inbreeding depression, genetic diversity, associative overdominance

## Abstract

Using computer simulation we explore the consequences of linkage on the inbreeding load of an equilibrium population, and on the efficiency of purging and the loss of genetic diversity after a reduction in population size. We find that linkage tends to cause increased inbreeding load due to the build up of coupling groups of (partially) recessive deleterious alleles. It also induces associative overdominance at neutral sites but rarely causes increased neutral genetic diversity in equilibrium populations. After a reduction in population size, linkage can cause some delay both for the expression of the inbreeding load and the corresponding purging. However, reasonable predictions can be obtained for the evolution of fitness under inbreeding and purging by using empirical estimates of the inbreeding depression rate. Purging selection against homozygotes for deleterious alleles affects the population’s pedigree. Furthermore, it can slow the loss of genetic diversity compared to that expected from the variance of gametic contributions to the breeding group and even from pedigree inbreeding. Under some conditions, this can lead to a smaller loss of genetic diversity, even below that expected from population size in the absence of selection.

The consequences of inbreeding on fitness are increasingly being recognized as a main factor potentially determining the extinction risk of endangered populations and, therefore, as a target to be controlled in conservation programs (Franklin 1980; Jamieson and Allendorf 2012; Frankham *et al.* 2014). It is now appreciated that, when a population experiences a reduction in size, any valid evaluation of its endangered status requires taking into account the inbreeding depression induced for fitness (Hedrick and Kalinowski 2000), but also that this implies considering the concurrent effects of genetic purging. In other words, it requires considering the boost of natural selection that is prompted by inbreeding as it exposes the recessive component of deleterious effects in homozygous genotypes (Leberg and Firmin 2008; Franklin *et al.* 2014; García-Dorado 2015).

Considerable progress has been attained in the comprehension and prediction of the consequences of genetic purging on average fitness through theoretical analysis (Glémin 2003; García-Dorado 2012) but, so far, these analyses are confined to the simplistic assumption of no linkage disequilibrium. In addition, purging can also affect the loss of genetic diversity caused by the reduction of population size, particularly in the presence of linkage. In general, natural selection against genetic variants at any given site is known to induce a reduction of effective population size at linked sites, so that tight linkage is expected to reduce the overall efficiency of natural selection in finite populations due to background selection, and to increase the rate of loss of genetic diversity (Hill and Robertson 1966; Nordborg *et al.* 1996; Santiago and Caballero 1998; Charlesworth 2012).

However, selection against recessive alleles induces some positive correlation between individual fitness and heterozygosity at neutral sites, known as associative overdominance (Ohta 1971) and, under very restrictive conditions, can cause some increase in neutral genetic diversity (Pálsson and Pamilo 1999). Furthermore, that correlation is known to be enhanced after the reduction of population size, mainly due to the increase of the variance for the inbreeding coefficient of individuals (individual inbreeding hereafter) (Bierne *et al.* 2000). This enhancement can also be accompanied by some delay in the loss of genetic diversity (Wang and Hill 1999; Wang *et al.* 1999). This should be ascribed to genetic purging which, operating on the basis of the association between individual fitness and individual inbreeding, affects the dynamics of neutral genetic diversity. Therefore, purging can be invoked as responsible for the observation that the loss of neutral genetic diversity during a sudden reduction of the population size is often smaller than expected (Rumball *et al.* 1994; Latter *et al.* 1995; Gilligan *et al.* 2005; Demontis *et al.* 2009; Turissini *et al.* 2014).

In this work, we investigate the consequences of linkage on inbreeding depression and purging, and on their impact upon genetic diversity. To achieve this, we use computer simulations to generate populations at the mutation-selection-drift balance for different values of the deleterious effect and dominance coefficient of mutations as well as for different genome lengths. Then, we derive lines that are maintained with reduced census size, and we analyze the consequences of inbreeding on the evolution of fitness and neutral genetic diversity. To disentangle the mechanisms responsible for the consequences of purging on genetic diversity, we track different measures of inbreeding. First, as a reference, we compute the inbreeding expected from the population size in the absence of selection under the classical ideal conditions (*i.e.*, the neutral prediction for Wright’s inbreeding coefficient *f*_N_). Furthermore, we compute the inbreeding expected from pedigrees (*f*_p_), and the inbreeding expected taking into account the variance of the number of gametes (*V*_k_) contributed by the individuals of the population to the next generation breeding pool (*f*_Vk_). Our results allow us to explore the inbreeding load concealed in the populations, its relationship with the population’s genetic diversity, and the mechanisms governing the evolution of both fitness and genetic diversity through generations after the reduction of census size.

## Materials and Methods

### Simulation of the base populations

Base populations of *N*_b_ = 1000 diploid individuals at the mutation-selection-drift (MSD) balance were obtained by simulating a life cycle consisting of mutation, selection, recombination and reproduction steps for 10,000 generations. Reproduction was panmictic, with selfing allowed. Each simulated haploid genome consisted of consecutive batches of 20 selective sites, followed by a marker neutral site, all along a single conceptual chromosome with length *L* Morgans. The numbers of neutral and selective sites simulated were *n*_n_ = 1000 and *n*_s_ = 20,000, respectively. The overall number of neutral or selective new mutations per generation was Poisson distributed with a mean of 0.1 deleterious mutations per haploid genome and generation, and 0.005 neutral mutations at neutral sites per haploid genome and generation. This implies the same average mutation rate per simulated site for markers and selective sites (5 × 10^−6^). New mutations were randomly assigned to the individuals and then to random nonsegregating sites. The segregating neutral sites should be interpreted as a sample of neutral marker segregating sites. The simulated selective sites can be interpreted as a small proportion of the actual number of sites where deleterious alleles might occur. However, the number of simulated selective sites was large enough to warrant that there were always nonsegregating sites to locate new deleterious mutations.

At selective sites, wild type alleles (+) mutated to deleterious alleles (*m*) with selection coefficient *s* and coefficient of dominance *h*, so that the three genotypes (++, +*m*, and *mm*) had fitness 1, 1 – *hs*, and 1 – *s*, respectively. Individual fitness (*w*_i_) was computed as the product of fitness values for all the *n*_s_ selective sites. To obtain each individual of the next generation, two parents were randomly sampled with replacement from the *N*_b_ individuals. The probability of each individual *i* of being sampled was proportional to its fitness *w*_i_. Then, a gamete was produced where the gene copy at the first site was chosen at random out of the two copies in the parent. For each consecutive site, the gene copy contributed to the gamete was sampled from the same chromosome or from the alternative one with probability (1 – *c*) or *c*, respectively, where c = 0.5 {1 – exp[−2L/(n_s_ + n_n_ − 1)]} represents the recombination fraction (Haldane 1919). Then, the two gametes produced were combined to form a new offspring individual; 50 base populations were simulated for each combination considered for deleterious effects (*s*, *h*) and genome length (*L*).

### Simulation of the reduced size lines

The above-mentioned MSD base populations were used to establish populations maintained with reduced size (hereafter referred to as lines). In each line, 100 individuals were produced each generation. New mutations were assigned to them and they were used to compute average fitness, as well as gene frequencies. Then, *N* individuals were randomly sampled as potential breeders, and the process was repeated for 100 generations.

Lines were maintained following the same mutation-selection-recombination-reproduction life cycle as for the base population (MSD scenario), or by avoiding natural selection [*i.e.*, all individuals had the same probability of being sampled as parents: mutation-drift (MD) scenario]. Pedigrees were saved, and the number of gametes *k* contributed by each potential breeder to the next generation was recorded to compute its corresponding variance (*V*_k_) each generation.

We analyzed a set of different cases aimed to qualitatively characterize the consequences of linkage on fitness and genetic diversity during genetic purging. These cases corresponded to all combinations for *s* = 0.1 or 0.5 with *h* = 0.5, 0.2, or 0 and free recombination or genome length *L* = 10, 5, or 1 Morgans. These specific parameters are not aimed at reproducing realistic rates of fitness declines but to expose and disentangle the mechanisms prompted by linkage under different situations. We explored the consequences of size reduction to *N* = 2, 10, or 50. For each base population, 1000 lines were derived for each reduced size (*N*) and scenario (MSD or MD) considered.

### Parameters assayed and estimates computed

At generation 10,000 of the base populations, the inbreeding load was computed as *B* = Σ 2*dq*(1 − q), where the addition is over sites, *q* is the frequency of the deleterious allele, and *d* *= s*(1/2 – *h*) is the purging coefficient (García-Dorado 2012). Linkage disequilibrium between two sites A and B, with mutant frequencies *q*_A_ and *q*_B_, respectively, was evaluated as

$$r_{\text{AB}}=\left[g\left(++\right)g\left(mm\right)-g\left(+m\right)g\left(m+\right)\right]/\surd \left[q_{\text{A}}\left(1-q_{\text{A}}\right)q_{\text{B}}\left(1-q_{\text{B}}\right)\right],$$ (1)

where (+*m*) stands for the frequency of a gamete composed of allele + in site A and allele *m* in site B, and so on (Hill and Robertson 1968). Considering only the set of segregating mutations, means for linkage disequilibrium were computed in the base populations by averaging *r*_AB_ over all values for adjacent selective sites (*r*).

Both at the base populations and for the derived reduced size lines, we computed the average fitness and the additive and dominance components of fitness genetic variance (Falconer and Mackay 1996). For the neutral sites, we computed the mean over lines for the actual heterozygosity of each individual *i* (*H_i_* = proportion of neutral sites that are heterozygous in individual *i*), its correlation with individual fitness within lines *r*(*w_i_*,*H_i_*), and the average genetic diversity [*H* = Σ 2*q*(1 − q)/*n*_n_, where the addition is over all *n*_n_ neutral sites].

The average inbreeding coefficient of the lines was computed for each generation *t* using different approaches:

- based on pedigree information (*f*_p_) using the tabular procedure (Emik and Terrill 1949);
- predicted from Wright’s equations using the number of potential breeders (Wright 1931), as
  $$f_{\text{N}}=1-{\left(1-1/2N\right)}^{t};$$ (2)
- predicted from Wright’s equations using the effective size (*N*_e_) expected on the basis of the variance (*V*_k_) of the number of gametes actually contributed by each individual to the next generation, and computed as
  $$f_{\text{Vk}}=1-{\left(1-1/2N_{\text{e}}\right)}^{t},$$ (3)

where *N*_e_ = (4*N* − 2) / (2 + *V*_k_) (Crow and Kimura 1970).

Since, in the absence of natural selection, the expected value of genetic diversity at generation *t* relative to that at the base population (*i.e.*, *H*_t_/*H*_0_), equals 1 – *f*_t_ (where *f*_t_ stands for Wright’s inbreeding coefficient), we plot *H*_t_/*H*_0_ through generations of reduced size against different estimates of the inbreeding coefficient in order to dissect the consequences of selection on genetic diversity.

The evolution of fitness after the reduction of population size was first predicted assuming relaxed selection, *i.e.*, using the classical neutral equation

$$w_{\text{t}}=w_{\text{0}}\exp\left(-\text{δ}f_{\text{N}}\right),$$ (4)

(Morton *et al.* 1956), where *w*_0_ and *w*_t_ are the average fitness value at the base population and at *t* generations with reduced size *N*, and *f*_N_ is the corresponding inbreeding coefficient computed from Equation 2. In the above expression, δ is the inbreeding depression rate (*i.e.*, the rate at which fitness should decline with increasing inbreeding under relaxed selection). For a single site model, or for a multisite model with no linkage disequilibrium, δ equals the inbreeding load *B*. Therefore, predictions from Equation 4 were obtained by replacing δ with the inbreeding load *B* of the base population. However, due to the multiplicative nature of fitness across different sites, the inbreeding depression rate in the early generations immediately after the reduction in population size is expected to be smaller (larger) than *B* when there is positive (negative) linkage disequilibrium between deleterious alleles at the base population. Therefore, predictions were also computed using an estimate of the inbreeding depression rate obtained by solving Equation 4 for δ using the fitness average obtained for *t* = 3 in the *N* = 2 lines maintained under the MSD scenario. This estimate of δ, analogous to the estimates that could be experimentally obtained in actual populations, was used to compute additional predictions of the evolution of fitness for *N* = 10 or *N* = 50 lines with Equation 4.

Predictions for the evolution of fitness in the lines were also obtained using the inbreeding-purging (IP) approach, by replacing *f*_N_ with the purged inbreeding coefficient *g_t_* (García-Dorado 2012) at generation *t*, computed as

$$g_{t}\approx \left[\left(1-1/2N\right)g_{t-1}+1/2N\right]\left(1-2d\;f_{N\;t-1}\right).$$ (5)

## Results

### The genetic properties of the base populations

Table 1 gives different genetic parameters assayed at the base populations after 10,000 simulated generations. Only scenarios of free recombination (FR) (along with their expectations, Exp) and close linkage (*L* = 1) are shown. Intermediate genome lengths gave intermediate results that are shown in Supporting Information, Table S1.

**Table 1:** Genetic parameters at the base populations

*s*	*h*	*d*	*L*	*w**^a^*	*B**^b^*	δ	*H**^c^*	*r*(*w*_i_, *H*_i_)	*LD* (*r* × 10^4^)	*c*
0.1	0.5	0.00	Exp	0.8187	0.0000		0.0196			
			FR	0.8189 ± 0.0014	0.0000 ± 0.0000		0.0195 ± 0.0004	0.0047	0.9583	0.5000 ± 0.0000
			1	0.8148 ± 0.0011	0.0000 ± 0.0000		0.0163 ± 0.0003	−0.0074	5.4753	0.0010 ± 0.0000
0.1	0.2	0.03	Exp	0.8187	0.2849		0.0196			
			FR	0.8221 ± 0.0011	0.2852 ± 0.0019	0.2807	0.0195 ± 0.0003	0.0055	−1.0360	0.5000 ± 0.0000
			1	0.8211 ± 0.0011	0.2867 ± 0.0018	0.2767	0.0160 ± 0.0003	0.0238***	8.7405***	0.0007 ± 0.0000
0.1	0.0	0.05	Exp	0.9048	2.3840		0.0196			
			FR	0.8972 ± 0.0011	2.5318 ± 0.0133	2.3199	0.0194 ± 0.0003	0.0216***	−2.0746*	0.5000 ± 0.0000
			1	0.8873 ± 0.0014	2.7576 ± 0.0170	2.0937	0.0178 ± 0.0003	0.1619***	23.7738***	0.0005 ± 0.0000
0.5	0.5	0.00	Exp	0.8187	0.0000		0.0196			
			FR	0.8200 ± 0.0017	0.0000 ± 0.0000		0.0176 ± 0.0003	0.0006	−1.8906	0.5000 ± 0.0000
			1	0.8190 ± 0.0011	0.0000 ± 0.0000		0.0156 ± 0.0003	0.0020	2.0133	0.0019 ± 0.0000
0.5	0.2	0.15	Exp	0.8187	0.2966		0.0196			
			FR	0.8205 ± 0.0014	0.2950 ± 0.0025	0.2833	0.0184 ± 0.0003	0.0058	1.2079	0.5000 ± 0.0000
			1	0.8178 ± 0.0012	0.3000 ± 0.0022	0.2848	0.0160 ± 0.0003	0.0021	3.9359	0.0013 ± 0.0000
0.5	0.0	0.25	Exp	0.9048	5.4865		0.0196			
			FR	0.8997 ± 0.0014	5.6341 ± 0.0277	5.3440	0.0193 ± 0.0004	0.0136	−0.4156	0.5000 ± 0.0000
			1	0.8870 ± 0.0016	6.5508 ± 0.0426	4.0936	0.0161 ± 0.0004	0.1072***	17.2425***	0.0005 ± 0.0000

Averages of different parameters (± SE) computed over 50 simulated base populations with different selection coefficients against homozygotes and dominance coefficients [*s* and *h*, respectively, with *d* *=* *s*(1/2 – *h*)] and different degrees of linkage (*FR* for free recombination, or genome length *L* = 1 Morgan). Parameters: average fitness (*w*); inbreeding load (*B*); inbreeding depression rate (δ) estimated using the average fitness obtained after three generations simulated with *N* = 2 in the MSD scenario; genetic diversity (expected heterozygosity) averaged over neutral sites (*H*); correlation coefficient between individual fitness and individual average heterozygosity [*r*(*w_i_*, *H_i_*)]; linkage disequilibrium (LD) averaged over all pairs of adjacent segregating selected sites (*r*); average recombination rate over all pairs of adjacent segregating selected sites (*c*). * and *** stand for significantly different from zero with *P*-value < 0.05 and 0.001, respectively, for sequential Bonferroni tests.

a Expected values (Exp) refer to the mutation-selection balance assuming no linkage disequilibrium (Crow and Kimura 1970).

b Expected values (Exp) refer to the mutation-selection-drift balance computed using the approximation by García-Dorado (2007), which assumes linkage equilibrium.

c Expected values (Exp) refer to the mutation-drift balance (Crow and Kimura 1970).

In the absence of linkage (FR), the observed inbreeding load (*B*) is very close to the theoretical prediction. Likewise, average fitness (*w*) and neutral genetic diversity (*H*) are very close to their corresponding expectations, and linkage disequilibrium is always nonsignificantly different from zero.

With reduced genome length, however, inbreeding load increases. This increase is only slight for *L* = 5 and *L* = 10 or for *h* = 0.2 (results not shown), but is more important for *h* = 0 and *L* = 1, particularly for the larger deleterious effect (*s* = 0.5). This increase of *B* is accompanied by an increase of coupling linkage disequilibrium (LD) between selective sites (*r* > 0) that also becomes relevant for *h* = 0, implying the presence of coupling groups of recessive deleterious alleles.

The correlation of neutral individual heterozygosity with individual fitness [*r*(*w*_i_,*H*_i_)], which measures associative overdominance, is always positive for *h* < 0.5 and increases as deleterious effects become more recessive and linkage becomes tighter. It is remarkable that, for small recessive deleterious effects (*s* = 0.1 and *h* = 0), this correlation becomes significantly positive even under free recombination. However, a shorter genome leads, in all cases, to reduced genetic diversity (*H*) in the base population, despite the increase in associative overdominance.

### The evolution of fitness in the lines

**Figure 1:**
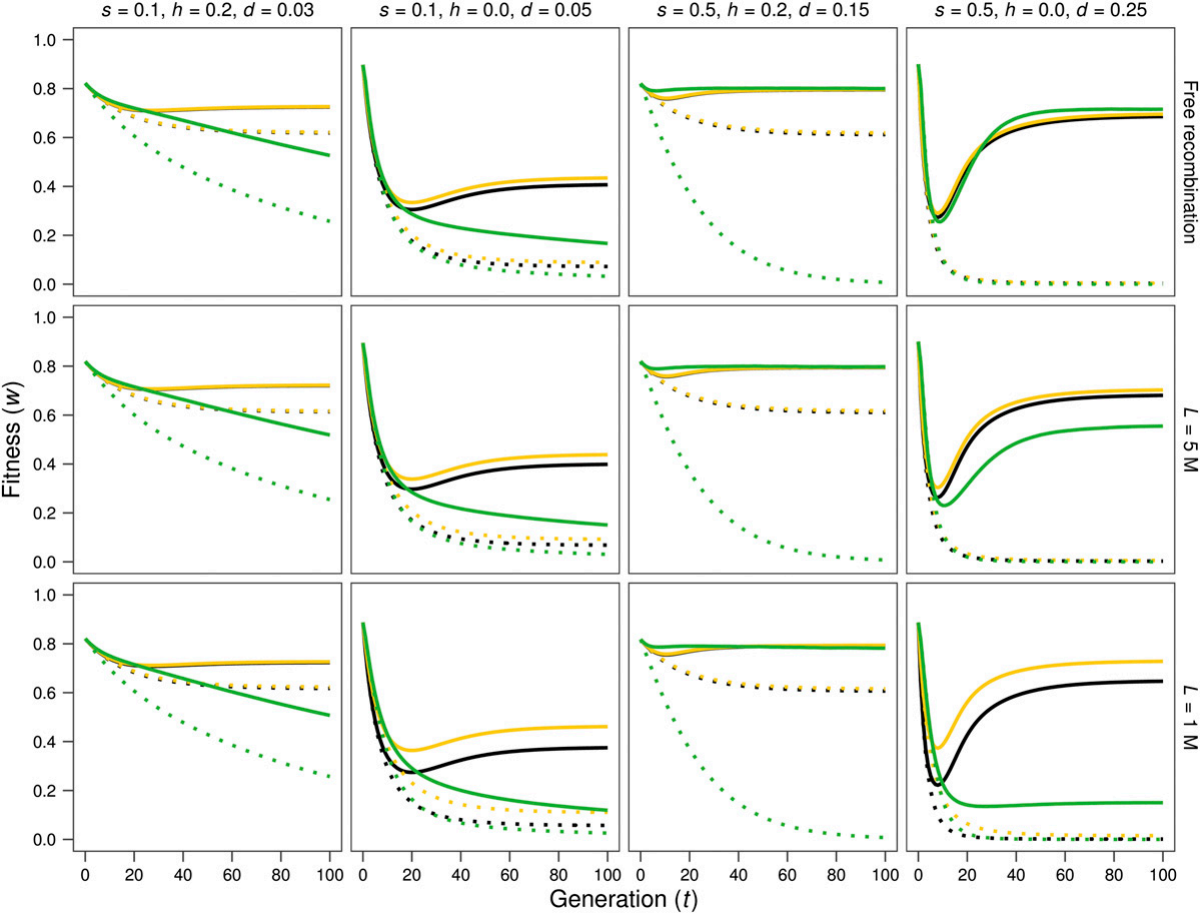
Evolution of average fitness after a reduction of population size to *N* = 10. Solid lines: mutation-selection-drift (MSD) results and inbreeding-purging (IP) predictions. Dotted lines: mutation-drift (MD) results and neutral predictions. Green: average fitness obtained from simulation results; black: average fitness predicted using the inbreeding load of the base population (*B*); yellow: average fitness predicted using the realized estimate of the inbreeding depression rate δ (see text). Note that these two predictions are on occasion so similar that the black line is completely hidden below the yellow one. Different panels represent different cases regarding the selection coefficient against homozygotes (*s*), the coefficient of dominance (*h*), the corresponding *d* value [*d* = *s*(1/2 – *h*)] and the genome length (*L*, given in Morgans).

**Figure 2:**
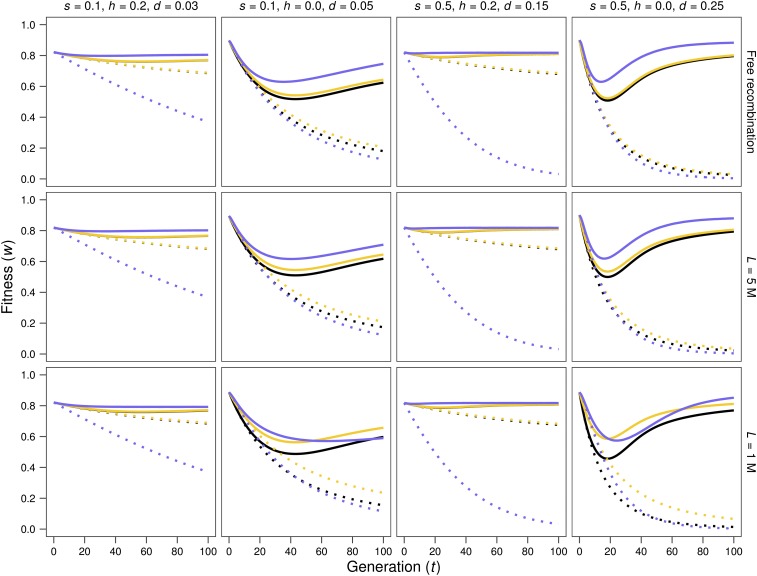
Evolution of average fitness after a reduction of population size to *N* = 50. Solid lines: MSD results and IP predictions. Dotted lines: MD results and neutral predictions. Blue: average fitness obtained from simulation results; black: average fitness predicted using the inbreeding load of the base population (*B*); yellow: average fitness predicted using the realized estimate of the inbreeding depression rate δ (see text). Note that these two predictions are on occasion so similar that the black line is completely hidden below the yellow one. Different panels represent different cases regarding the selection coefficient against homozygotes (*s*), the coefficient of dominance (*h*), the corresponding *d* value [*d* = *s*(1/2 – *h*)] and the genome length (*L*, given in Morgans).

**Figure 3:**
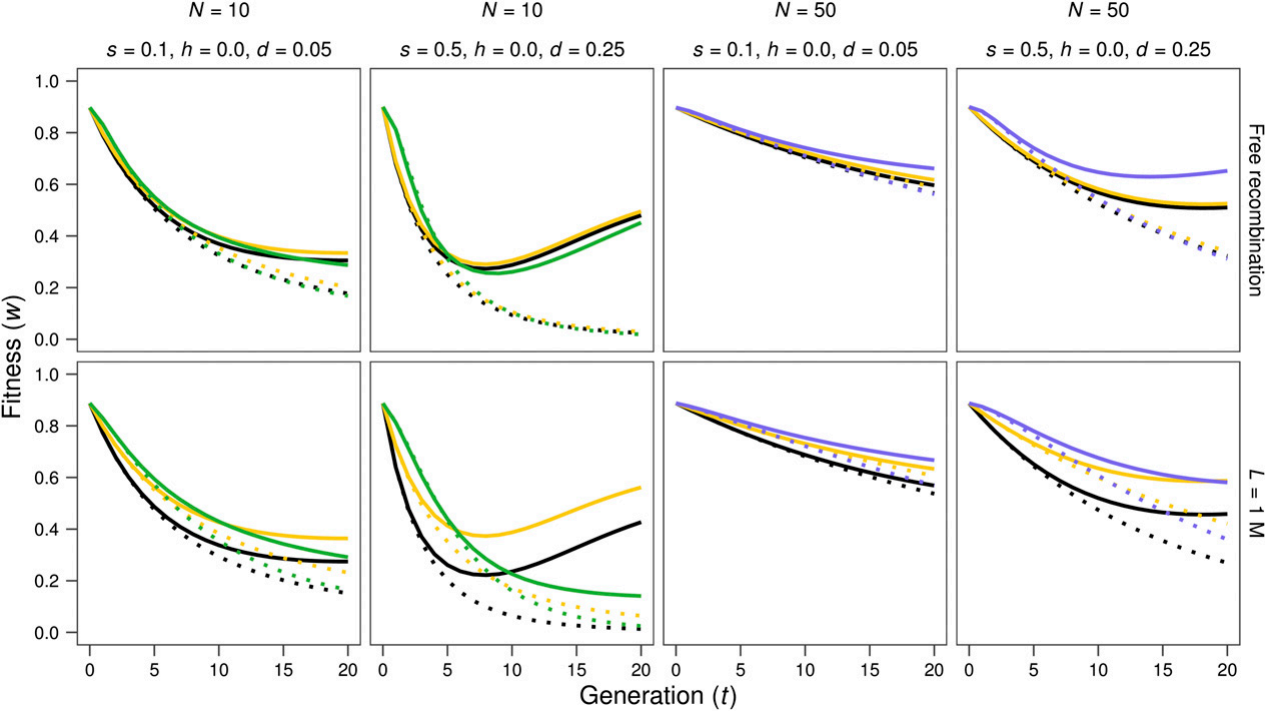
Short-term evolution of average fitness after a reduction of population size. Solid lines: MSD results and IP predictions. Dotted lines: MD results and neutral predictions. Green: average fitness obtained from simulation results for *N* = 10; blue: average fitness obtained from simulation results for *N* = 50; black: average fitness predicted using the inbreeding load of the base population (*B*); yellow: average fitness predicted using the realized estimate of the inbreeding depression rate δ (see text). Different panels represent different cases regarding *d* value [*d* = *s*(1/2 – *h*)] for *h* = 0, genome length (*L*, given in Morgans), and reduced census (*N*).

Figure 1 and Figure 2 give the evolution of fitness through generations after size reduction starting from base populations with inbreeding load (*h* < 0.5). Simulations are represented by solid (MSD scenario) or dotted (MD scenario) lines, in green color for *N* = 10 in Figure 1, and blue color for *N* = 50 in Figure 2, as in the remaining figures. Neutral predictions (dotted lines) and IP predictions (continuous lines), computed using the inbreeding load obtained in the base populations (*B*; in black) or the inbreeding depression rate estimated from simulations (δ; in yellow), are also represented. For *N* = 2, fitness decline is very similar under the MSD and the MD scenarios (not shown), since purging becomes inefficient as *Nd* drops below 1. Therefore, Figure 1 and Figure 2 show results just for *N* = 10 and *N* = 50 cases. Figure 3 shows in more detail the short-term evolution for some of the cases above.

During the first generations after the reduction in size (Figure 3), both the IP predictions and the evolution of fitness observed under the MSD scenario (continuous lines) are quite similar to neutral predictions based on the δ value (yellow dotted lines, where δ had been estimated from the fitness decline in the MSD lines with *N* = 2 at generation three). The smaller *N*, the fewer generations lasts this similitude; say three generations for *N* = 10 and about six generations for *N* = 50. Thus, estimates of the inbreeding depression rate obtained after a few generations of fast inbreeding provide reliable and conservative predictions of the inbreeding depression for less drastic reductions in size.

However, Figure 1 and Figure 2 show that, in the absence of linkage, fitness average in the MSD scenario becomes well above the neutral prediction after a few generations. They confirm that, for *Nd* > 1, purging becomes increasingly efficient with increasing *d* values. In addition, new deleterious mutation causes important fitness decline in the MD scenario and, in the long term, also some decline in the MSD one for *Ns* < 1, as expected.

Both under free recombination and in the presence of linkage, IP predictions tend to be conservative (*i.e.*, to predict a fitness average smaller than that observed), at least in the short-to-medium term. This is particularly so when predictions are computed using *B*, but also when obtained from δ (green or blue lines *vs.* black and yellow solid lines in Figure 1, Figure 2, and Figure 3). Nevertheless, for small *Ns* values, the long-term decline can become considerably larger than the corresponding IP predictions, as expected from accumulation of new deleterious mutations *(N* = 10 with *s* = 0.1 in Figure 1).

**Figure 4:**
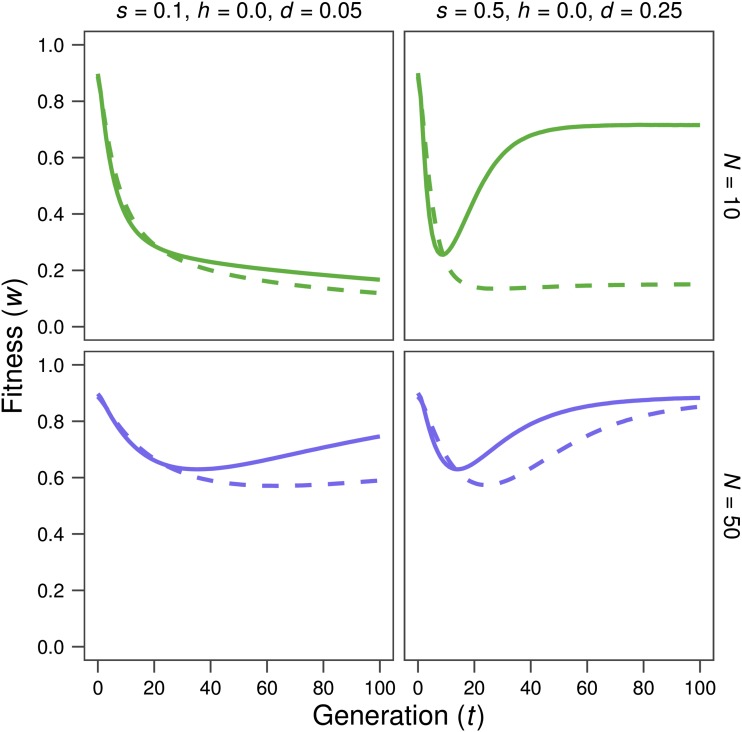
Evolution of average fitness after a reduction of population size (N = 10, green; N = 50, blue) under no recombination or tight linkage. Simulation results for the decline of fitness under free recombination (solid lines) or a genome length of *L* = 1 Morgan (dashed lines).

In the presence of linkage, and for the cases with *h* = 0, which are those inducing significant positive linkage disequilibrium between selected sites in the base population, the δ estimate (*i.e.*, the inbreeding depression rate computed from the early inbreeding rate in *N* = 2 lines under the MSD scenario) drops for shorter genomes (Table 1), while the inbreeding load (*B*) increases. Thus, δ can become considerably smaller than *B*. Furthermore, the IP prediction computed using *B* overrates the short-term fitness decline for short genomes, even in cases where it predicts very accurately those obtained under free recombination. This can be seen from Figure 3 by comparing the fitness averages of simulated lines (green or blue solid lines) *vs.* the corresponding predictions (black solid lines) for *L* = 1. Since the slowdown of early fitness decline in shorter genomes is also observed in the MD scenarios (compare dotted green or blue lines *vs.* dotted black lines in Figure 3), it should not be ascribed just to purging, but to the fact that the full expression of the inbreeding load concealed in the base population requires the destruction through generations of the coupling disequilibrium between deleterious alleles, due to recombination. As a result, despite the larger inbreeding load (*B*) concealed at the MSD balance for shorter genomes, early fitness decline under reduced census is quite similar to that obtained in the absence of linkage and, occasionally, can even be slightly smaller, as illustrated in Figure 4, which represents the fitness decline observed under the MSD scenario for free recombination (continuous line) and tight linkage (*L* = 1; broken line).

Nevertheless, tight linkage causes some reduction in the efficiency of purging, due to background selection. Thus, for *Nd* not much larger than 1, purging can be relatively efficient under free recombination but can become inefficient for shorter genomes, as illustrated for the medium- or long-term fitness decline in the cases with *Nd* = 2.5 (*d* = 0.25 for *N* = 10 or *d* = 0.05 for *N* = 50 in the upper right and lower left graphs in Figure 4, respectively). It is worth noting that this consequence of linkage is not observed for the case *d* = 0.15 with *N* = 10 (third column of graphs in Figure 1), suggesting that it depends on the linkage disequilibrium generated in the base population (see Table 1). However, as long as *Nd* is well above 1, purging remains quite efficient despite linkage and, overall, neither the initial fitness decline nor the long-term fitness values are substantially affected by linkage, although the decline phase can last longer, and the minimum value attained for fitness can be smaller (Figure 4).

### The evolution of inbreeding and neutral genetic diversity in the lines

In what follows, unless otherwise stated, we will refer to simulation results obtained after the reduction of size for lines maintained with natural selection (MSD scenario), although we will occasionally refer to those obtained under the MD scenario for comparison.

For the additive cases (*h* = 0.5), *f*_p_ increases slightly faster than in the MD scenario, as expected from the increase in the variance of gametic contributions (*V*_k_) induced by natural selection. Furthermore, *f*_p_ and *f*_Vk_ increase virtually at the same rate, even though the latter has been computed ignoring the heritable component of *V*_k_. These observations are not affected by genome length (Figure S3 and Figure S6). The decline of neutral genetic diversity is slightly faster than in the MD scenario, due to the same cause. This phenomenon is more obvious for short genomes, since natural selection is known to reduce the effective population size for neutral sites closely linked to selected ones (Figure S9). All these effects are, however, very slight, because, for *h* = 0.5, additive variance for fitness is very small in the base population at the MSD balance (it is in fact very close to the 2λ*hs* value expected at the mutation-selection balance; Figure S19, Figure S20, and Figure S21), leading to a small intensity for natural selection, both in the base populations and after size reduction.

In what follows we analyze the consequences of purging on the evolution of different estimates of inbreeding and on the loss of neutral genetic diversity. For *h* = 0.2 no relevant consequences were detected, as results were similar to those for *h* = 0.5. Therefore, we will concentrate on the cases of completely recessive deleterious mutation (*h* = 0). Analogous results for *h* = 0.2 and *h* = 0.5 can be found in Figure S1, Figure S2, Figure S3, Figure S4, Figure S5, Figure S6, Figure S7, Figure S8, Figure S9, Figure S10, Figure S11, Figure S12, Figure S13, Figure S14, Figure S15, Figure S16, Figure S17, and Figure S18).

**Figure 5:**
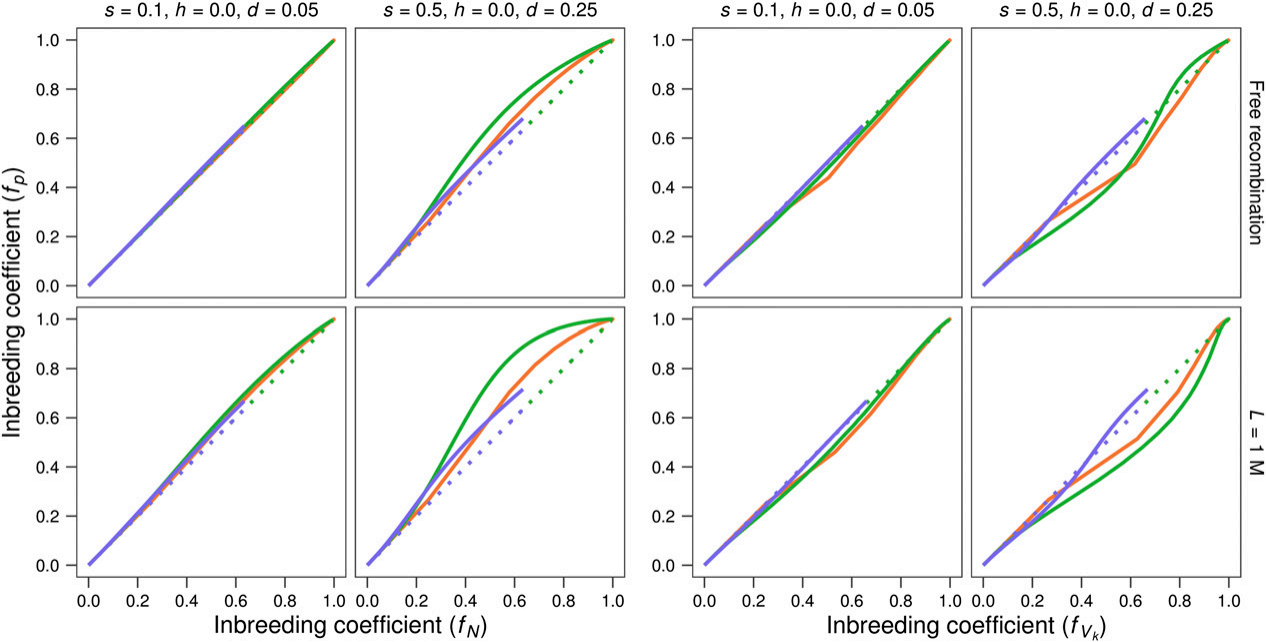
Average pedigree inbreeding plotted against inbreeding expected just from the census or taking into account the variance of gametic contributions. Per-generation average inbreeding obtained from pedigrees in the lines (*f*_p_) represented for two different deleterious effects (*s*) with completely recessive gene action (*h* = 0), and assuming free recombination or a genome length of *L* = 1 Morgan. Results are plotted against the inbreeding expected from the census (*f*_N_) in the four left panels, and against the inbreeding expected taking into account the observed variance of gametic contributions (*f*_Vk_) in the four right panels. Red: *N* = 2; green: *N* = 10; blue: *N* = 50. Solid lines: lines maintained under the MSD scenario. Dotted lines: lines maintained under the MD scenario.

Figure 5 (four left panels) shows that, for *h* = 0, pedigree inbreeding increases faster than the neutral prediction, as expected due to natural selection. However, the four right panels show that this increase of pedigree inbreeding (*f*_p_) is slower than that of *f*_Vk_, which implies that the individuals showing larger average coancestry with the individuals of the line contribute on the average fewer gametes to the next generation. This disadvantage for individuals with larger average coancestry is not observed in the MD scenario, and should therefore be ascribed to purging.

**Figure 6:**
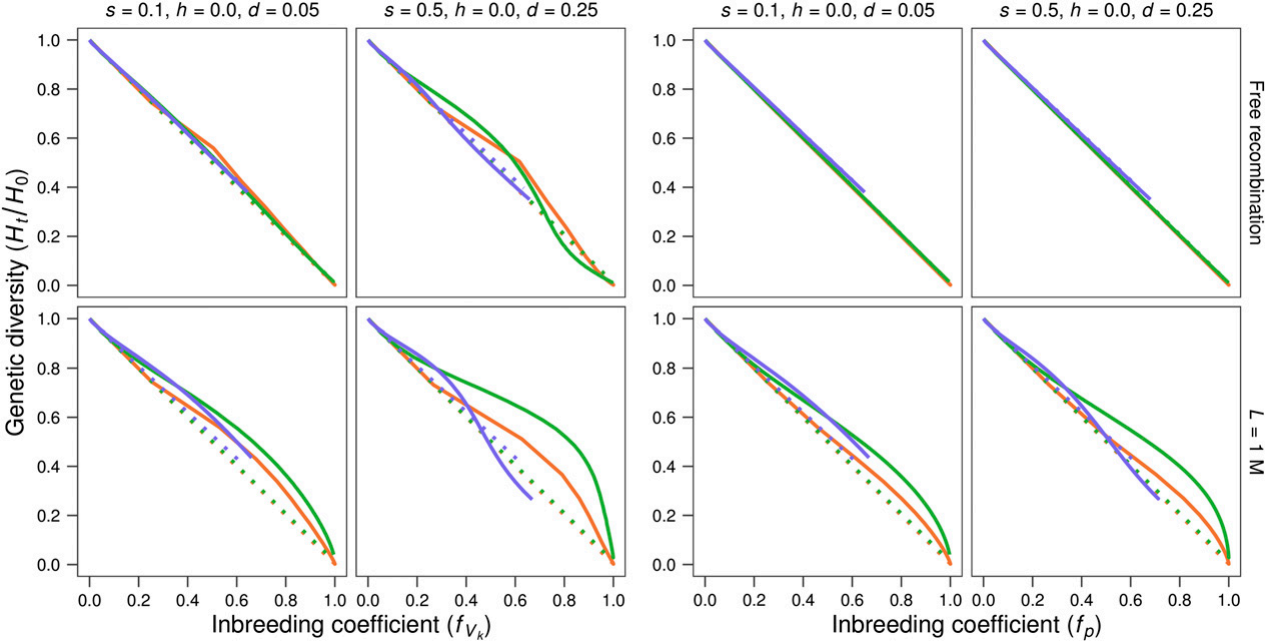
Relative neutral genetic diversity plotted against *f*_Vk_ or *f*_p_. Per-generation average of relative neutral genetic diversity in the lines (*H*_t_/*H*_0_) plotted against the corresponding inbreeding expected taking into account the observed variance of gametic contributions (*f*_Vk_) or the inbreeding computed using pedigree information (*f*_p_), (four left or four right panels, respectively). The different panels correspond to different deleterious effects (*s*) with completely recessive gene action (*h* = 0), assuming free recombination or a genome length of *L* = 1 Morgan. Red: *N* = 2; green: *N* = 10; blue: *N* = 50. Solid lines: lines maintained under the MSD scenario. Dotted lines: lines maintained under the MD scenario.

Figure 6 (four left panels) shows that neutral genetic diversity relative to the ancestral value (*H*_t_/*H*_0_) declines more slowly than expected from *f*_Vk_ (*i.e.*, it takes values above the diagonal). This observation, more conspicuous in the presence of linkage, could in principle be ascribed to the increased contribution of individuals with smaller average coancestry noted above. However, the four right panels of Figure 6 show that, in the presence of linkage, *H*_t_/*H*_0_ drops even more slowly than expected from pedigree inbreeding, (*i.e.*, it takes again values above the diagonal in Figure 6, mainly for *L* = 1), a behavior also restricted to the MSD scenario and, therefore, ascribed to the joint effect of linkage and purging selection, rather than to new mutation.

**Figure 7:**
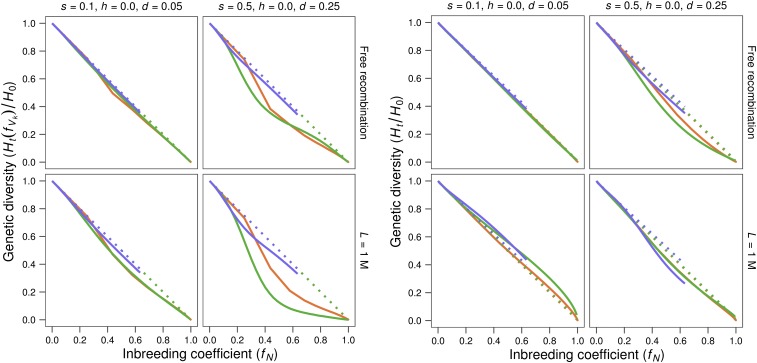
Relative neutral genetic diversity against inbreeding expected just from the census. The four left panels show the relative neutral genetic diversity expected in the lines on the basis of the observed variance of gametic contributions [*H*_t_(*f*_Vk_)/*H*_0_]. The four right panels show the observed relative neutral genetic diversity. In both cases, relative neutral genetic diversity is plotted against the corresponding inbreeding expected from the census (*f*_N_). The different panels correspond to two different deleterious effects (*s*) with completely recessive gene action (*h* = 0), and assume free recombination or a genome length of *L* = 1 Morgan. Red: *N* = 2; green: *N* = 10; blue: *N* = 50. Solid lines: lines maintained under the MSD scenario. Dotted lines: lines maintained under the MD scenario.

We have computed the proportion of the genetic diversity of the base population that is expected to persist through the period of maintenance with reduced size taking into account the observed values for *V*_k_. This expected proportion, denoted by *H*_t_(*f*_Vk_)/*H*_0_ [where *H*_t_(*f*_Vk_) stands for the prediction of *H*_t_ computed as a function of *f*_Vk_], is plotted in Figure 7 (four left panels) against the inbreeding expected when all individuals have the same reproductive opportunities [*i.e.*, this figure plots (1 – *f*_Vk_) *vs. f*_N_]. It shows that the expected neutral genetic diversity computed taking into account the variance of gametic contributions (*V*_k_) caused by purging, can be much smaller than under nonpurging (*i.e.*, can give lines well below the diagonal). In contrast, the four right-hand panels of Figure 7 show that the actual reduction of *H*_t_/*H*_0_ *vs. f*_N_ is not too different from that expected in the absence of purging, given by the diagonal. In fact, although *H*_t_/*H*_0_ usually declines at a rate somewhat larger than in the neutral nonpurging model, there is even one tight linkage case (*s* = 0.1, *h* = 0, *L* = 1) where the decline of neutral genetic diversity is considerably slower than expected under drift alone. This is not observed for the analogous case with larger deleterious effects (*s* = 0.5, *h* = 0, *L* = 1), probably because there are fewer segregating deleterious alleles and linkage between them is therefore weaker.

In summary, the loss of genetic diversity due to genetic purging is smaller than expected from $f_{V_{K}}$ and, in the presence of linkage, it can also be, to some extent, smaller than expected from pedigree inbreeding, *f*_p_. Occasionally, purging can cause even slower decline of genetic diversity than expected from population size in the absence of purging (*i.e.*, that predicted from *f*_N_), although this was only detected for tight linkage. It should be noted that these observations hold even when drift is so intense that purging is unable to hold back inbreeding depression, as occurs for *N* = 2. Thus, purging can slow the loss of genetic diversity even in situations in which it is not able to slow the rate of fitness decline.

## Discussion

Our simulation results show that panmictic populations at the MSD balance can conceal larger inbreeding load in the presence of linkage than under free recombination. This is due to the build up of coupling groups of deleterious alleles, and requires the presence of linked segregating sites with deleterious alleles that are substantially recessive. In principle, natural selection against linked additive deleterious alleles is expected to induce negative linkage disequilibrium in finite populations (Barton and Otto 2005). However, our results show that selection against recessive alleles can induce the opposite effect. The reason is that each recessive deleterious allele of a coupling group has an advantage when in heterozygous condition (compared to an uncoupled deleterious copy), as it is more likely to be in an individual that is also heterozygous for the remaining alleles of its group. Of course, it also suffers from some disadvantage when homozygote, by being in individuals that are more likely also to be homozygotes for the remaining alleles of their group, but still the resulting expected fitness can be higher than that corresponding to an uncoupled deleterious copy. Thus, coupled deleterious alleles are partially sheltered against natural selection, leading to some increase of the inbreeding load. It should, however, be noted that this did not lead to any increase of the expressed load (*i.e.*, the average fitness of the panmictic base populations shows no appreciable reduction).

After a reduction in population size, inbreeding exposes the recessive component of deleterious effects (cases with *h* < 0.5). Therefore, it induces a genetic excess for the variance of the gametic contributions to the next generations (*V*_k_), compared to an ideal situation where all individuals have identical fitness. This excess determines the intensity of purging selection, while purging efficiency could be quantified by the reduction it causes of the fitness decline induced by inbreeding. Thus, the per-generation efficiency of purging could be defined as the difference between the per-generation decline expected just from inbreeding and that realized under inbreeding and purging (*i.e.*, the net increase of fitness ascribed to purging). Therefore, according to Fisher’s theorem of natural selection, in the absence of drift, the efficiency of purging per generation should equal the additive component of the increase in *V*_k_ caused by inbreeding (both additive and dominance components are plotted against generation number in Figure S19, Figure S20, Figure S21, Figure S22, Figure S23, and Figure S24).

In the absence of linkage, our results confirm that IP predictions for the efficiency of purging are conservative for *Nd* > 1, *i.e.*, they predict a somewhat higher inbreeding depression than it is observed. They also confirm that purging efficiency increases with *d*, as expected. These findings are in agreement with previous results (Pérez-Figueroa *et al.* 2009).

In the presence of linkage, although the base population can conceal more inbreeding load due to positive linkage disequilibrium, the actual inbreeding depression rate during the early phase after the reduction of the population size is smaller than *B*, even in the absence of purging. The reason for this can be illustrated by considering a couple of linked loci (A and C). In our multiplicative fitness model, the average fitness at generation *t* (with inbreeding *f*_t_) is:

$$\text{E}\left(w_{\text{t}}\right)=\text{E}{\left(w_{\text{A}}w_{\text{C}}\right)}_{t}=\text{E}{\left(w_{\text{A}}\right)}_{\text{t}}\text{E}{\left(w_{\text{C}}\right)}_{\text{t}}+\text{Cov}{\left(w_{\text{A}},w_{\text{C}}\right)}_{\text{t}},$$

where E stands for expectation, *w*_A_ stands for the fitness of individuals al locus A, and Cov stands for covariance. When A and C are associated in a coupling group, the covariance term is positive, so that the expected fitness at generation *t* is larger than in the absence of linkage disequilibrium, and the realized rate in inbreeding depression is therefore smaller. This explains why the larger value of *B* maintained in the presence of linkage, does not translate into a higher rate of fitness decline in the short–medium term, since the full expression of the inbreeding load requires the progressive destruction of the initial linkage disequilibrium due to recombination. Nevertheless, purging can become less efficient in small genomes for values of *Nd* close to 1, due to background selection (Charlesworth 2012). In contrast, when *Nd* is substantially above 1, overall purging efficiency is scarcely affected by genome length, although both inbreeding depression and purging can be delayed.

Selection against partially recessive deleterious alleles also affects the neutral genetic diversity expected at the MSD balance. In principle, in the absence of dominance, a reduction of neutral diversity is expected from natural selection, due to a reduction in the effective size (Santiago and Caballero 1998). However, it has been appreciated that selection against (partially) recessive deleterious alleles can increase genetic diversity in closely linked neutral sites. Thus, Pálsson and Pamilo (1999) reported higher genetic diversity at neutral sites when they were tightly linked (*c* = 10^−5^) to selected sites with *Nhs* ≤ 4, although inspection of their figure 1f reveals that increased neutral genetic diversity also requires that *Nhs* is much smaller than *Ns* (*i.e.*, large *d*) and, of course, it requires *Ns* > 1. Our *h* = 0 cases fulfill these conditions regarding *Nhs* and *Ns*, but, even for *L* = 1, each neutral mutation had one recessive deleterious allele to each side at an average distance about 5 × 10^−4^ M, *i.e.*, about 50 times larger than in the case analyzed by Pálsson and Pamilo (1999). In our evaluated scenarios, neutral genetic diversity is always smaller than its neutral expectations, suggesting that selection against (partially) recessive deleterious alleles only leads to increased neutral genetic diversity in equilibrium populations under very restrictive conditions, including a genome densely covered by segregating sites with largely recessive deleterious alleles.

Despite this, we found that natural selection against (partially) recessive deleterious alleles produces in the base populations a positive association between average fitness and heterozygosity at neutral sites, *i.e.*, associative overdominance. To understand the reasons for this association, it is convenient to consider individual inbreeding at the base MSD balance population, as it could be defined by reference to any arbitrary generation in the recent past. Then it is obvious that individuals with larger inbreeding are expected to have both smaller fitness (due to inbreeding depression) and smaller neutral genetic diversity, which prompts a positive correlation between the two latter variables [*i.e.*, *r*(*w*_i_,*H*_i_) *>* 0]. Thus, the fact that associative overdominance is observed even in the absence of linkage, should be ascribed to the association between individual heterozygosity on neutral and selected sites caused by mating of related individuals in finite populations. Furthermore, we observe that *r*(*w*_i_,*H*_i_) increases for reduced genome length, because linkage implies an association for the heterozygosity at different sites in the offspring of each single mating. Note, however, that associative overdominance does not imply increased neutral genetic diversity. These observations are in agreement with Ohta’s (1971) consideration that

It is possible that the associative overdominance is ineffective as a mechanism for the maintenance of genetic variabilities, except for some special cases such as inversion chromosome and semi-isolated local populations. Its real importance lies in the fact that it is probably responsible for most of the observed superiority of heterozygotes.

In fact, it could be more illuminating to consider associative overdominance not as a mechanism at all, but just as a side effect created by selection when it is constrained to operate mostly against homozygotes: an illusion of advantage for heterozygotes that, occasionally, concurs with some increase of neutral diversity. The dependence of natural selection against recessive alleles on inbreeding is known to be able to induce some increase in neutral genetic diversity at equilibrium populations, as it imposes restrictions on the evolution of genealogical relationships between individuals and of the probability of identity by descent at neutral sites closely linked to selected ones. Nevertheless, the conditions for this phenomenon, including close linkage and largely recessive deleterious effects, are so restrictive that they were never met in our panmictic base populations at the MSD balance, where neutral genetic diversity was never larger than that expected in the neutral model and did not increase with associative overdominance.

However, as a drastic reduction of population size induces a high inbreeding rate, it also increases the magnitude of the negative covariance between identity by descent and fitness. Therefore, as purging acts upon fitness, it can slow the progress of pedigree inbreeding below that expected from the variance of gametic contributions, as observed for *h* = 0. In fact, the delay of *f*_p_ compared to *f*_Vk_ (Figure 5) should be even larger than computed here, as the rate of increase of *f*_Vk_, considering long-term contributions rather than one generation contributions, would be larger than that given by Equation 3 due to the heritable component of the variance of gametic contributions.

This delay of *f*_p_ compared to *f*_Vk_ implies that purging against recessive deleterious alleles produces an advantage for individuals showing smaller average coancestry, and, therefore, tends to equalize the contributions of the different founding individuals to future generations in a way that is, to some extent, analogous to the minimum coancestry strategy proposed in conservation programs to reduce the loss of genetic diversity in endangered populations (Ballou and Lacy 1995; Sonesson and Meuwissen 2002; Fernández *et al.* 2003). To understand this process, it is useful to consider a reduced size population at the time it is derived from the ancestral population, and to imagine that every founder individual carries ancestral recessive deleterious alleles that are different from those carried by the remaining founders. In such a situation, purging, by opposing the increase in frequency that would be expected from genetic drift for some of these deleterious alleles, will prevent excessive contribution from any founder individual to future generations. Figure 5 (right) shows that this phenomenon is somewhat more intense for shorter genomes, which conceal larger inbreeding loads (Table 1).

Furthermore, for neutral sites closely linked to selected ones (Figure 6), the loss of genetic diversity is even slower than expected from pedigree inbreeding, in agreement with previous findings by Wang *et al.* (1999), who also described slowed loss of neutral diversity under full sib mating in the presence of recessive alleles with moderate deleterious effect. This implies that some additional mechanism, operating only in the presence of linkage, contributes to slow down the loss of neutral genetic diversity. Since partial equalization of contributions from founders is already accounted for by using *f*_p_, this additional mechanism should involve some equalization of the contribution to future generations of the two haploid sets of neutral alleles for each founder individual. Again, to visualize this mechanism, we can think of a situation where the two haploid sets of each single founder individual carry different recessive deleterious alleles. Then purging, when preventing the overrepresentation in future generations for each ancestral deleterious allele, will favor equal representation of both haploid sets for selected sites, preserving genetic diversity for neutral sites linked to them.

The more common consequence of the two equalization processes described above is a loss of genetic diversity smaller than would be expected from standard selection of similar intensity but, usually, around that expected from genetic drift in the absence of selection. As this loss of genetic diversity can be smaller than that expected from pedigree inbreeding, this process could be responsible for the slowed loss of genetic diversity that has often been observed experimentally (Rumball *et al.* 1994; Latter *et al.* 1995; Gilligan *et al.* 2005; Demontis *et al.* 2009; Turissini *et al.* 2014).

In fact, the genetic diversity at the neutral sites can occasionally decline during purging even more slowly than expected from the population size in the absence of selection, although this occurred under restrictive conditions, involving tight linkage (Figure 7). Thus, this phenomenon is observed only for relatively small recessive deleterious effects, suggesting that it requires some compromise between intense purging and the existence of many sites segregating for deleterious alleles in the base population, so that each individual carries several alleles to be purged and there is on average tight linkage between purged alleles and neutral ones. This would favor the equalization of the ancestral neutral copies to future generations.

Thus, two main lessons should be drawn from the above analysis that can be useful to conservation practices. The first concerns fitness inbreeding depression. Although, for small genome lengths, the large ancestral population can conceal larger inbreeding load than under free recombination, this does not imply increased rates of inbreeding depression in the short term, so that the inbreeding depression rate can be empirically estimated from short-term inbreeding depression, and the evolution of fitness after a reduction in population size can still be reasonably predicted from IP theory. Obviously, this depends on the availability of a reliable estimate of the purging coefficient (Bersabé and García‐Dorado 2013). Nevertheless, for genomes with strong linkage, somewhat larger population sizes can be needed to warrant that purging is able to overcome genetic drift so that IP predictions remain conservative.

The second lesson has to do with the decision about whether or not managing the gametic contributions. This management can be as simple as the equalization of the number of offspring contributed by each parent to the next generation breeding pool, or can be intended to minimize the average coancestry in the offspring in order to equalize the contributions of the founder individuals (Sonesson and Meuwissen 2002; Fernández *et al.* 2003). Regarding the reduction of fitness inbreeding depression, managing gametic contributions is to be preferred when population size is so small that purging is expected to be inefficient (García-Dorado 2012), and also, in the short term, when, in the light of the population’s reproductive potential, early inbreeding depression is expected to pose an excessive risk before purging becomes efficient (García-Dorado 2015). However, managing gametic contributions is also advised in order to minimize the loss of genetic diversity. Our results show that, when considering this latter motivation, it should be taken into account that purging itself tends, to some extent, to equalize the contributions of ancestral nonpurged alleles. Although the rate of loss of genetic diversity expected under purging selection is likely to be larger than that expected under direct management of gametic contributions, it can be well below that expected from the intensity of natural selection and even below that expected from the apparent inbreeding estimated from genealogy. On occasion, this consideration may tip the scale in favor of a nonmanagement strategy.
